# Nivolumab‐induced celiac‐like enteropathy in patient with metastatic renal cell carcinoma: Case report and review of the literature

**DOI:** 10.1002/ccr3.2342

**Published:** 2019-07-28

**Authors:** Lucie Duval, Sarah Habes, Thierry Chatellier, Pascale Guerzider, Céline Bossard, Claude Masliah, Isabelle Archambeaud, Yann Touchefeu, Tamara Matysiak‐Budnik

**Affiliations:** ^1^ IMAD, Hepato‐Gastroenterology & Digestive Oncology Hôtel Dieu, CHU de Nantes Nantes France; ^2^ University of Nantes Nantes France; ^3^ Clinic of Estuaire Saint Nazaire France; ^4^ Department of Pathology Hospital of Saint Nazaire Saint Nazaire France; ^5^ Department of Pathology University Hospital of Nantes Nantes France; ^6^ INSERMU1235, TENS University Bretagne Loire Nantes France

**Keywords:** celiac‐like enteropathy, immune‐related adverse effects, immunotherapy, nivolumab, PD‐1 inhibitors

## Abstract

Nivolumab may induce severe celiac‐like enteropathy, that may appear very rapidly, after only two injections of nivolumab, and may be successfully treated with corticosteroids. This observation underlines the importance of histological analysis of duodenal biopsies and the necessity to rule out a real celiac disease in patients with nivolumab‐induced diarrhea.

## 1 INTRODUCTION

Immunotherapy using monoclonal antibodies targeting the programmed death‐1 receptor (PD‐1) and its ligand (PD‐L1) has become a standard of care for an increasing number of cancers. Nivolumab, an anti‐PD‐1 monoclonal antibody, has shown its efficacy in improving survival in patients with metastatic renal cell carcinoma (RCC),^1^, ^2^ and has been approved as a second‐line treatment, after the failure of angiogenesis‐targeted therapy, in this indication. Even though nivolumab has a relatively good tolerance profile, it may induce severe immunologic toxicities that can occur at any time during or even after the treatment, and these side effects are of crucial importance for patient's care.^3^, ^4^, ^5^, ^6^ Immune‐related gastrointestinal toxicities are among the most frequently observed and are dominated by colitis, while small intestine toxicities are much less frequent.^7^, ^8^ Only one case of diffuse enteropathy, with immune infiltration extended from the duodenum to the colon, has been reported after a long‐term course of nivolumab in a patient with lung cancer.^9^ Here we report a case of severe celiac‐like enteropathy, without colitis, induced by nivolumab in patient with metastatic RCC. To our knowledge, this is the first published case of this type of enteropathy induced by nivolumab.

## 2 CASE REPORT

A 58‐year‐old male patient, with a medical history of insulin‐requiring diabetes and hypercholesterolemia, was diagnosed with RCC. In February 2010, the patient underwent left nephrectomy for the tumor classified pT3bNxM0. No adjuvant treatment was administered and the patient was followed up with a chest‐abdomen‐pelvis CT Scan (CAP‐CT) every 3 months. In November 2011, hepatic and pancreatic metastases appeared. In April 2012, the patient underwent a caudal spleno‐pancreatectomy and cholecystectomy, followed by radiofrequency ablation of the unique hepatic metastasis located in the segment VII. He was considered in complete remission and was followed up with CAP‐CT every 6 months. In 2014, recurrence of the disease was diagnosed at several locations: right kidney, adrenal glands, lumbar‐aortic lymph nodes, and lungs. According to the standard of care, a treatment by sunitinib (Sutent^®^) was introduced in August 2014, at the dose of 50 mg/d during 4 consecutive weeks followed by a 2‐week break. The treatment was poorly tolerated, marked by asthenia, skin, thyroid, and renal toxicities, motivating the reduction of dose to 37.5 mg/d. In 2017, a right testicular metastasis was discovered and orchiectomy was performed. The CAP‐CT performed in January 2018, showed the progression of target lesions estimated at 30% according to the RECIST criteria. It was decided to stop the treatment by sunitinib and to introduce immunotherapy by nivolumab. The first IV injection of nivolumab at a standard dose of 3 mg/kg (240 mg) took place on February 9, 2018, and the treatment was scheduled for one IV infusion every 2 weeks.

In March 2018, after two injections of nivolumab, the patient presented biological signs of diabetic decompensation and of hyperthyroidism, leading to the cessation of treatment. He did not have diarrhea or any other overt clinical symptoms at that time. The TSH level was low (0.06 µU/mL), the antithyroperoxidase and antithyroglobulin antibodies were positive, and the thyroid scintigraphy was not in favor of Basedow disease, thus reinforcing hypothesis of autoimmune thyroiditis induced by nivolumab.

In April 2018, 1 month after the last injection of nivolumab, the patient was admitted again to the hospital because of vomiting, diarrhea, and weight loss of 3 kg in 2 weeks. A recto‐sigmoidoscopy and oeso‐gastroduodenal endoscopy were macroscopically normal and systematic biopsies were obtained. The CAP‐CT showed some visible mesenteric lymph with moderate overall distension of the small bowel loops. Stool culture was positive for *Arcobacter butzleri*, and it was decided to introduce antibiotic treatment with azithromycin and fluoroquinolones. The general status of the patient deteriorated, with signs of septic shock and identification of bacteremia with *Citrobacter freundii*, and the patient was transferred to the intensive care unit where he stayed from the 9th to 14th of May 2018. Initially, the clinical evolution was favorable on antibiotherapy (Ceftriaxone and Ornidazole) and the introduction of small doses of norepinephrine. After a few days, however, the patient's state deteriorated and he developed severe anorexia, vomiting, and profuse, especially nocturnal diarrhea, complicated by malnutrition requiring parenteral nutrition. The blood tests showed the biological signs of malabsorption with profound hypoalbuminemia (15.5 g/L, N > 37), decreased serum level of iron (0.52 mg/L, N > 7.5) and folic acid (2.3 ng/mL, N > 5), hypomagnesemia (0.53 mmol/L, N > 0.7), and hypocalcemia (1.89 mmol/L, N > 2.2). A second CAP‐CT was performed on the May 29, 2018 and showed the same aspect of the overall distension of the small bowel loops.

**Figure 1:**
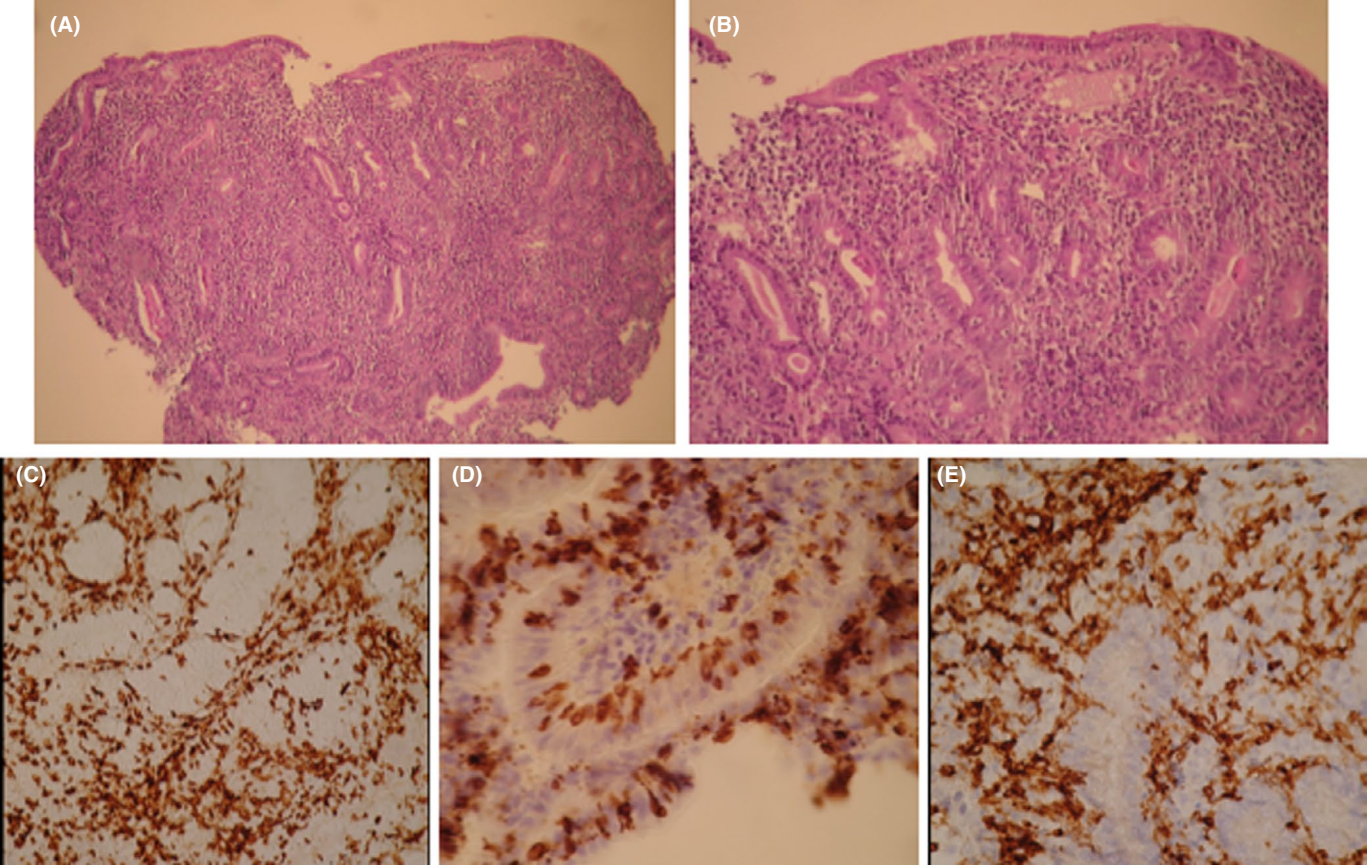
Histological sections of the duodenal mucosa obtained in May 2018, at the time of severe diarrhea. Staining with hematoxylin & eosin, showing a total villous atrophy, crypt hyperplasia and numerous lymphocytes in the lamina propria (A, magnification ×100, B, magnification ×250). Immunostaining with anti‐CD3 (C), anti‐CD8 (D), anti‐CD4 (E), showing a high number of intraepithelial CD3+ and CD8+ lymphocytes, and a dense infiltrate with CD3+ and CD4+ lymphocytes in the lamina propria (magnification ×400)

A second endoscopic evaluation was performed with a complete ileo‐colonoscopy to eliminate macroscopic colitis, and it was macroscopically and microscopically normal (both, colonic and ileal biopsies). At the same time, the results of histological analysis of the duodenal biopsies obtained during the initial endoscopic examination were available and they showed a normal histology of gastric and colonic mucosa. However, the duodenal biopsies showed a subtotal villous atrophy with chronic duodenitis, with an abundant lymphocyte CD3+, CD4+, CD8+ infiltration and intraepithelial lymphocytosis (Figure 1). The antitransglutaminase and antigliadin antibodies were negative, not in favor of celiac disease. The gluten free diet was introduced but had no effect on symptoms.

Due to the oral alimentation intolerance, the parenteral nutrition was continued. Since the enteral immune toxicity was suspected, a treatment with parenteral methylprednisolone (2 mg/kg/d), was started. After only 2 days of treatment, the symptoms improved with a spectacular decrease in stool frequency. Intravenous methylprednisolone treatment was continued for 5 days and then maintained orally at the dose of 100 mg/d. The patient was discharged from the hospital on a gradually decreasing dose of corticosteroids during a 12‐week period, after which he was placed on hydrocortisone 20 mg daily due to his history of adrenalectomy.

**Figure 2:**
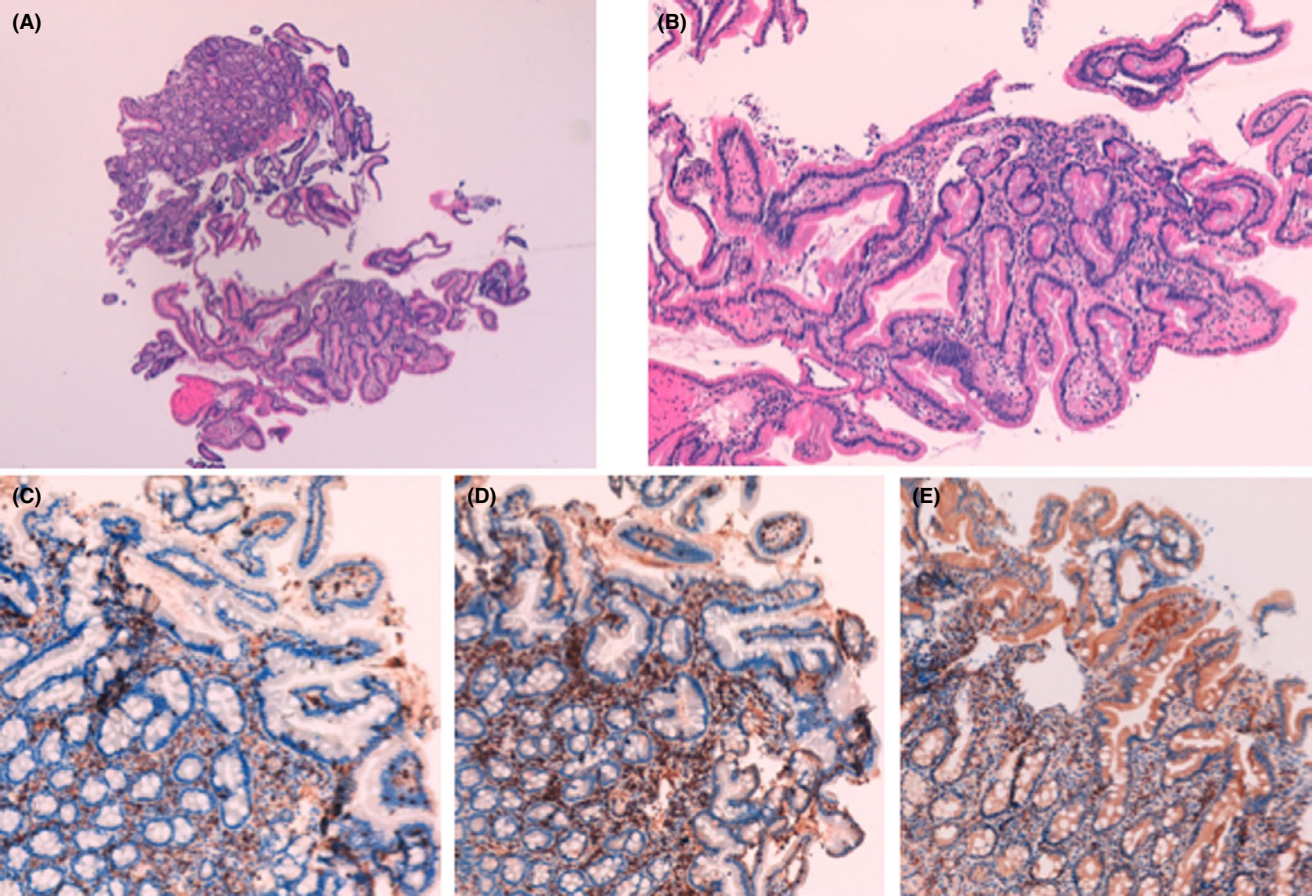
Histological sections of the duodenal mucosa obtained in February 2019, 10 mo after cessation of treatment with nivolumab and 6 mo after complete resolution of clinical symptoms. Staining with hematoxylin & eosin, showing a duodenal mucosa with a normal architecture and differentiation, and a normal density of lymphocytes, both in lamina propria and in epithelium (A, magnification ×50; B, magnification ×100). Immunostaining with anti‐C3 (C), anti‐CD8 (D), anti‐CD4 (E), showing a normal B and T lymphocyte distribution and count, both in lamina propria and in the epithelium (magnification ×100)

Four months after his discharge from the hospital (10th of October 2018), the patient was in a good general status, had no digestive symptoms, his biological tests were normal, and he was still in therapeutic pause for his RCC since the CT Scan was showing a stability of the disease. In December 2018, a new evolution of the tumor disease was observed, motivating the introduction of treatment by cabozantinib. The treatment is well tolerated, marked only by slight asthenia. In February 2019, a control upper endoscopy was performed to verify the evolution the duodenal lesions and confirmed a complete normalization of duodenal histology (Figure 2).

## 3 DISCUSSION AND CONCLUSIONS

The immune check point inhibitors (ICPI) have revolutionized the treatment of patients with several cancers, but at the same time, they have brought a new type of cancer treatment‐related side effects, named immune‐ related adverse effects (IRAE). Currently, two major types of ICPI are used, the antibodies blocking the cytotoxic T‐ lymphocyte antigen 4 (CTLA‐4; ipilimumab, tremelimumab) and those blocking the programmed death 1 (PD‐1) receptor or its ligand (PD‐L1; nivolumab and pembrolizumab, or atezolizumab and durvalumab). This blockage results in reactivation of cytotoxic T cells which can destroy tumor cells, but may also compromise immune‐tolerance and lead to the development of autoimmune manifestations.

The IRAE can affect several organs including endocrine system, skin, digestive system, kidneys, peripheral and central nervous systems, eyes, and others,^3^, ^10^ and may appear in a wide variety of forms ranging from mild to severe, and even fatal.^11^, ^12^ Digestive toxicities are among the most frequently reported and are dominated by diarrhea and colitis, both more frequently observed during the treatment with anti‐CTLA‐4 ‐ than anti‐PD‐1 agents.^5^, ^8^, ^13^ Although colitis is the most frequent digestive toxicity of ICPI, enterocolitis, with clinical features of inflammatory bowel disease, has also been observed.^14^

One systematic review of all case reports of IRAE published until 2016, described 251 cases, most of them treated with ipilimumab and only seven patients treated with nivolumab. The most frequent toxicities were digestive, dominated by colitis and enterocolitis (30%), all appeared in patients treated with ipilimumab, while no digestive toxicities were observed in patients treated with nivolumab or pembrolizumab.^15^

More recently, few case reports of digestive toxicity induced by nivolumab, including colitis or enterocolitis, have been published.^16^, ^17^, ^18^, ^19^ Our case differs from the other published cases by several features: (a) the toxicity appeared very rapidly, only after two infusions of nivolumab, (b) this toxicity was mainly limited to the proximal part of the small intestine (duodenum), there were no signs of macro‐ or microscopic colitis, (c) the histological type of lesions was also very particular, “celiac‐like,” characterized by villous atrophy and intraepithelial lymphocytosis, typical for active celiac disease, but with no other features of CD, and especially with negative celiac serology.

So far, only one case of nivolumab‐induced enteropathy has been published, and concerned a young female patient treated with nivolumab for NSCLC who developed, after the 18th nivolumab infusion, a severe diffuse enteropathy with villous atrophy, associated with collagenous colitis and negative celiac disease serology.^9^ This case differs from ours by the fact that the symptoms appeared after a long‐term treatment with nivolumab, and the lesions were more diffuse, involving not only the small intestine but also the colon, revealing a diffuse autoimmune inflammation of the small and large intestine.

Celiac‐like enteropathy seems to be an exceptional complication of ICPI, and has been mainly described in patients treated with anti‐CTLA‐4 agents.^15^ In 2013, one case of a “real” celiac disease (CD) revealed by treatment with ipilimumab was reported in a patient with prostate cancer.^20^ The patient was first treated by budesonide, but then could be managed only on a gluten free diet. This case demonstrates that ipilimumab may reveal a real CD, although it is not certain whether it amplified the symptoms of already existing CD or induced de novo CD. Indeed, the implication of CTLA‐4 in CD has already been suggested.^21^, ^22^ The hypothetical mechanism could be that the soluble CTLA‐4 may have an immunomodulatory effect on T cells and CTLA‐4 inhibition may lead to the up‐regulation of T cell response, susceptible to reveal CD.^20^, ^21^ Such a mechanism has not been proposed for PD‐1 inhibitors, but since they also induce T‐cell up‐regulation, it is not unlikely that the similar mechanism may play a role. It seems probable that PD‐1 inhibitors‐induced gastrointestinal toxicity results from the interaction between the genes, the environment, the immune system and microbiome.^23^

It should be also reminded that celiac‐like enteropathy has been described for other drugs like sartans, and in particular for olmesartan.^24^ In a French series of 36 patients, olmesartan‐associated enteropathy was frequently (but not always) associated with villous atrophy, negative antitransglutaminase and antienterocyte antibodies, but positive antinuclear antibodies, suggesting that also in this case immunological mechanisms may play a role.^25^

## 4 CONCLUSION

We report here an exceptional case of severe nivolumab‐ induced celiac‐like enteropathy, appeared only after two infusions of nivolumab, well responding to systemic corticosteroid treatment. This report, together with the data from the literature, may suggest the indication of systematic testing for celiac disease in all patients before introducing the treatment with ICPI.

## CONFLICT OF INTEREST

None declared.

## AUTHOR CONTRIBUTIONS

LD, SH, TC, CM and IA: were key clinical members for patient care. PG, CB: performed histological analysis and provided histological images. LD, YT and TMB: performed literature review and were major contributors in manuscript preparation. All authors contributed to critical revision of the manuscript. All authors approved the manuscript for submission.
